# The MODY-Causing Mutation of the Human Carboxyl Ester Lipase Gene (*CEL*) Triggers Chronic Pancreatitis but not Diabetes in Mice

**DOI:** 10.1053/j.gastro.2025.01.243

**Published:** 2025-02-14

**Authors:** ANNY GRAVDAL, STEVEN J. WILHELM, CEL-MODY MOUSE PROJECT, MARK E. LOWE, ANDERS MOLVEN, XUNJUN K. XIAO, KARIANNE FJELD

**Affiliations:** The Gade Laboratory for Pathology, Department of Clinical Medicine, *and* Mohn Center for Diabetes Precision Medicine, Department of Clinical Science, University of Bergen Bergen, Norway, *and* Department of Medical Genetics, Haukeland University Hospital, Bergen, Norway; Department of Pediatrics, Washington University School of Medicine, St Louis, Missouri; Department of Pediatrics, Washington University School of Medicine, St Louis, Missouri; The Gade Laboratory for Pathology, Department of Clinical Medicine, University of Bergen, Bergen, Norway, *and* Department of Pathology and Section for Cancer Genomics, Laboratory Clinic, *and* Department of Pathology, Haukeland University Hospital, Bergen, Norway; Department of Pediatrics, Washington University School of Medicine, St Louis, Missouri; The Gade Laboratory for Pathology, Department of Clinical Medicine, *and* Mohn Center for Diabetes Precision Medicine, Department of Clinical Science, University of Bergen, Bergen, Norway, *and* Department of Medical Genetics, Haukeland University Hospital, Bergen, Norway

Carboxyl ester lipase (CEL) is a digestive enzyme expressed by pancreatic acinar cells. The last exon of the *CEL* gene contains a variable number tandem repeat (VNTR).^1^ Single-base deletions within the *CEL* VNTR cause maturity-onset diabetes of the young, type 8 (MODY8 or *CEL*-MODY), an inherited syndrome of pancreatic exocrine and endocrine dysfunction.^2^ Families with single-base deletions in the first, fourth, or fifth VNTR repeat have been described.^2,3^ These mutations lead to frameshifts that result in misfolding, aggregation, and impaired secretion of the aberrant CEL protein,^3,4^ promoting endoplasmic reticulum (ER) stress and apoptosis through a maladaptive unfolded protein response.^4^

Previous attempts to model MODY8 in mice were unsuccessful as both *Cel* knock-out mice^5^ and a transgenic model expressing the mutated *CEL* variant^6^ had normal pancreatic phenotype. Here we report a knock-in MODY8 animal model (denoted *Cel-MODY*) where we substituted the three-repeat mouse *Cel* VNTR with the disease-causing *CEL* VNTR of MODY8 patients. To control whether VNTR expansion is pathologic, we created a parallel mouse strain (*Cel-16R*) carrying the normal 16-repeat VNTR of human *CEL*.

We first tested cellular expression of the two mouse carboxyl ester lipase (mCEL) proteins harboring human VNTRs, denoted mCEL-MODY and mCEL-16R. Compared to wild-type mCEL and mCEL-16R, more mCEL-MODY retained as insoluble and less was secreted (Supplementary Figures 1A through 1C), similar to the pathogenic human CEL variants.^4^ *Cel-MODY* and *Cel-16R* mice were then created (see Methods). We confirmed pancreas expression of the “humanized” mCEL proteins by immunoblotting of tissue extracts from both mice strains (Supplementary Figure 1D). Because MODY8-affected subjects are heterozygous for mutation, we initially focused our study on heterozygous mice (*Cel*^+*/MODY*^) from 1 through 12 months of age. The animals bred normally. As no phenotypic differences were observed between males and females, we present results for male mice only.

Bodyweights of *Cel*^+*/MODY*^, *Cel*^+*/16R*^ and *Cel*^+*/*+^ mice were similar at ages 1 through 12 months. However, *Cel*^+*/MODY*^ mice exhibited reduced pancreas weight and spontaneously recapitulated histologic features of chronic pancreatitis including pancreatic atrophy, inflammation, and fatty changes (Figures 1A and 1B). Histopathologic scoring showed a progressive pancreatic phenotype with age in *Cel-MODY* mice and no discernible histologic changes for *Cel-16R* (not shown). Pancreatitis was confirmed in *Cel*^+*/MODY*^ animals by increased serum amylase, fibrosis, and macrophage infiltration (Figures 1C through 1F).

Pancreatic function was evaluated by measuring digestive enzyme activity. Total lipase activity was elevated at 3 months and reduced at 12 months of age in *Cel*^+*/MODY*^. Amylase activity was reduced at 6 and 12 months of age. Trypsin levels tended to be lower in *Cel*^+*/MODY*^ than in controls (Figure 1G). These results are consistent with impaired pancreatic exocrine function as *Cel-MODY* mice age, most likely by a mechanism not involving intra-pancreatic trypsin activation.

To exclude the possibility that pancreatitis initiation might depend on an overactive CEL enzyme, we created a *Cel-MODY* mouse with a mutation (S194A) in the active site of CEL. The S194A mutation inactivated mCEL catalytic activity but had no effect on the cellular partition of the mutant protein (Supplementary Figures 1E through 1H). *Cel*^+*/MODY*^-S194A mice had the same pancreatic phenotype as *Cel*^+*/MODY*^ mice, whereas *Cel*^+*/*+^-S194A mice exhibited normal pancreatic morphology (Supplementary Figure 1I). These findings support our hypothesis that MODY8 pathogenicity is rooted in proteotoxicity and not aberrant CEL activity.

Immunohistochemistry of pancreatic sections from *Cel-MODY* mice revealed more intense CEL signals with darker, irregular puncta than in controls (Figure 1H). These puncta were seen both intra- and extracellularly in the exocrine pancreas, and in areas with acinar atrophy and low CEL expression. For assessing ER stress, we measured pancreatic mRNA expression of *Hspa5*, *Ddit3,* and the unfolded protein response target genes *Aft3, Aft4, Aft6,* and *Xbp1.* For all markers, there was a trend of upregulation in *Cel*^+*/MODY*^ mice with significant increases of *Hspa5* (binding immunoglobulin protein [*BiP*]) and *Ddit3* (*Chop*) (Figure 1I), and *Aft3, Aft4 and Aft6* (Supplementary Figure 2A). Immunostaining for BiP in *Cel*^+*/MODY*^ mice and a MODY8 patient displayed widespread and stronger staining in atrophic areas compared to normal pancreatic tissue (Figure 1J). Moreover, acinar cell ultrastructure showed dilated ER and mitochondrial shape changes in *Cel*^+*/MODY*^ mice (Figure 1K).

*Cel-MODY* mice also recapitulated radiographic features of the MODY8 pancreas. When homozygous 6-month-old mice were evaluated by abdominal magnetic resonance imaging, T1-/T2-weighted coronal series depicted coarse texture of the pancreas and reduced organ size, very similar to what is reported for MODY8 patients (Figures 1L and 1M).^2,3^ Microscopically, homozygous mice had nearly identical pancreatic morphology as heterozygotes did, but the pathology progressed faster and was more prominent. In some animals, the atrophic pancreas contained macroscopically visible cysts (Figure 1L), again akin to the human MODY8 phenotype where cysts are commonly found.^3^

Diabetes is a hallmark of human MODY8.^2,3^ Still, we observed no impaired glucose homeostasis in *Cel*^+*/MODY*^ mice compared to *Cel*^+*/16R*^ and *Cel*^+*/*+^ animals. We measured random blood glucose biweekly at 2 to 12 months, performed intraperitoneal glucose and insulin tolerance tests at 6 and 12 months, and tested homozygous animals at 6 months. No matter the timepoint, experiment, or genotype, there was no sign of elevated blood glucose in *Cel-MODY* mice (Supplementary Figures 2B through 2F). Intriguingly, the *Cel-MODY* animals demonstrated somewhat better glucose tolerance than controls.

The islets of Langerhans were evaluated by histology and immunostaining. We observed a significant increase in islet number and area in 12-month-old *Cel-MODY* mice (Supplementary Figure 2G). The islets’ content of alpha cells and Ki67-positive beta-cells was not significantly different between the two mouse genotypes (Supplementary Figures 2H through 2J). Our observations suggest that the animals increase islet cell mass in response to the *Cel-MODY* mutation and then mostly by beta-cell hypertrophy.

In conclusion, *Cel-MODY* mice fully recapitulate the exocrine phenotype of human MODY8 and provide strong evidence for a proteotoxic etiology. Why our model has no endocrine phenotype is unclear. *Cel-MODY* islets were negative for CEL staining (not shown), suggesting that islet uptake of aggregated CEL protein does not happen. We did not expose our animals to additional endocrine stressors, such as high-fat diet or streptozotocin, which could have revealed a phenotypic difference. However, mouse models for pancreatitis generally do not lead to diabetes development,^7^ and one obvious explanation is species-specific differences between mouse and man.^8^ Removal of up to 90% of rodent pancreas does not affect glucose homeostasis, suggesting a larger reserve capacity than in humans.^9^ Still, cellular transplantation experiments have shown that some endocrine aspects of MODY8 can be recapitulated in mice.^10^ This raises interesting questions about human-mouse differences and exocrine-endocrine crosstalk that should be the focus of further investigations.

## Supplementary Material

1

## Figures and Tables

**Figure 1.**
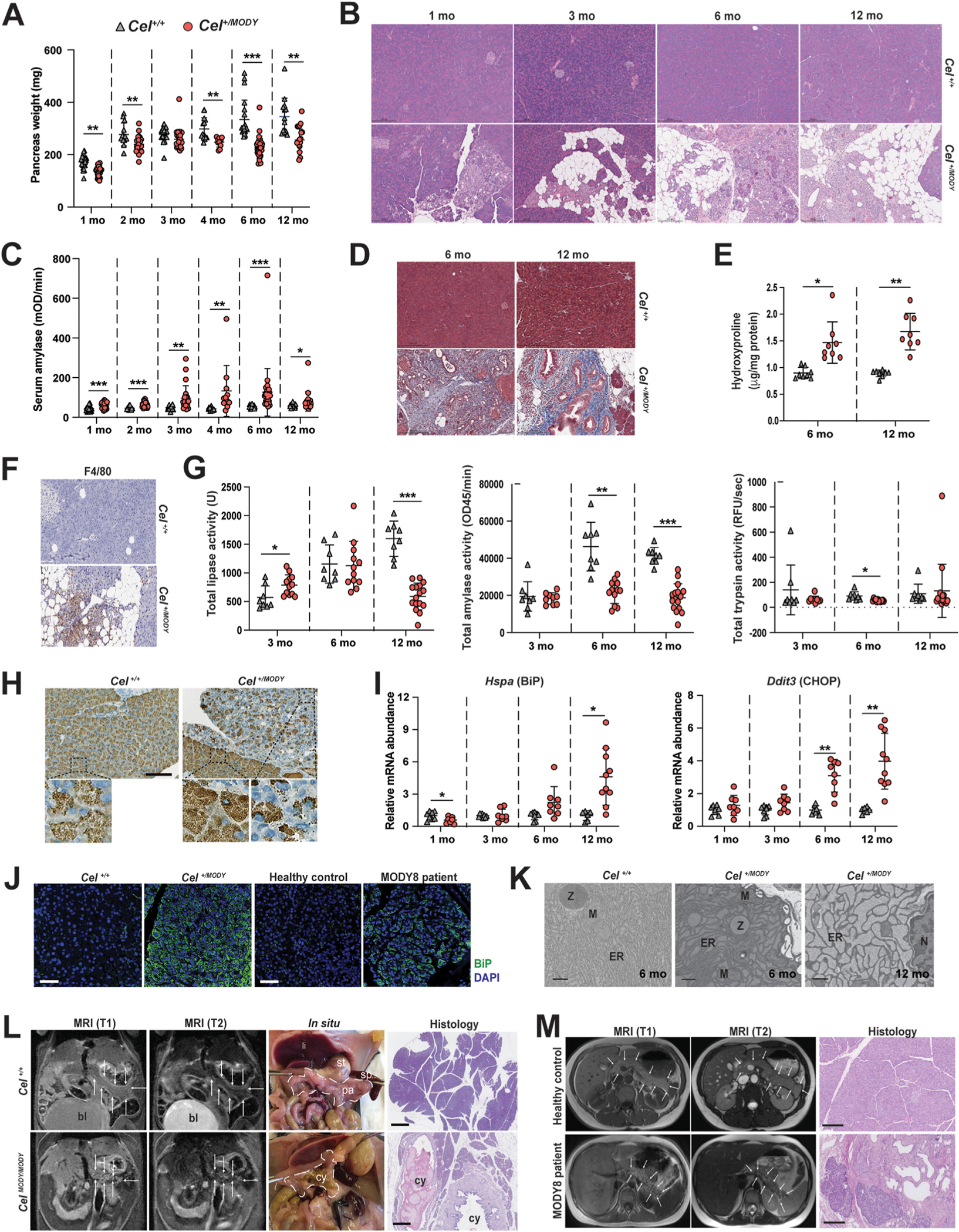
Pancreas pathology in carboxyl ester lipase (CEL) maturity-onset diabetes of the young (*Cel-MODY*) mice. Control (*Cel*^+*/*+^), heterozygous (*Cel*^+*/MODY*^) and homozygous (*Cel*^*MODY/MODY*^) male mice were analyzed as depicted in the panels. Human samples were included in panels *J* and *M*. (*A*) Pancreas weight at age 1, 2, 3, 4, 6, and 12 months. (*B*) hematoxylin and eosin (H&E)–stained pancreas sections from mice at age 1, 3, 6, and 12 months. Scale bars are 200 *μ*m. (*C*) Serum amylase activity at age 1, 2, 3, 4, 6, and 12 months. (*D*) Pancreas sections from mice at age 3, 6, and 12 months stained by Masson’s trichrome stain. (*E*) Hydroxyproline content in pancreatic tissue extracts (at age 6 and 12 months). (*F*) Chromogenic staining for the macrophage marker F4/80 in pancreas sections (age 12 months). (*G*) Intrapancreatic lipase, amylase and trypsin activity at age 3, 6, and 12 months. For each enzyme, total pancreatic activity was calculated. (*H*) Chromogenic staining of CEL protein in pancreas sections (age 6 months). The lower panels are higher magnifications of the rectangles in the upper panels. Scale bar is 300 *μ*m. (*I*) mRNA levels of endoplasmic reticulum (ER) stress markers *Hspa5* (encoding binding immunoglobulin protein [BiP]) and *Ddit3* (encoding CHOP) at age 1, 3, 6, and 12 months. (*J*) Fluorescent immunostaining of the BiP protein (*green*) in representative pancreas sections from *Cel*^+*/*+^ and *Cel*^+*/MODY*^ mice (age 6 months), a healthy human control, and a maturity-onset diabetes of the young, type 8 (MODY8) patient. 4’,6-diamidino-2-phenylindole (DAPI) staining of nuclei is in *blue*. Scale bars are 50 *μ*m. (*K*) Transmission electron microscopy of pancreas sections. Scale bars are 500 nm. *(L)* Pancreatic structure in *Cel-MODY* mice. Abdominal MRI was performed at age 6 months. Coronal T1- and T2-weighted magnetic resonance imaging (MRI) shows the pancreas with its boundaries demarcated by *white arrows*. In the in situ images, the boundaries of the pancreas are marked with a *dashed white line*. In each row, the MRI, in situ, and pancreas histology images are from one representative mouse, either a *Cel*^+*/*+^ control mouse (*upper panel*) or a *Cel*^*MODY/MODY*^ mouse (*lower panel*). Scale bars are 500 *μ*m. *(M)* Pancreatic structure in human MODY8. Axial T1- and T2-weighted MRI depicting the pancreas with its boundaries demarcated by *white arrows* in a healthy individual (*upper panel*) and a MODY8 patient (*lower panel*). H&E sections from a healthy individual and a MODY8 patient are presented to the *right*. Scale bars are 250 mm. In panels *A, C, E, G* and *I*, individual values with mean (horizontal bar) ± SD are shown. bl, bladder; cy, cyst; ER, endoplasmic reticulum; li, liver; M, mitochondria; N, nucleus; pa, pancreas; sp, spleen; st, stomach; Z, zymogen granules. *P≤ .05; **P≤ .001; ***P≤ .0001.

## Data Availability

Original data and materials are available from the authors upon reasonable request.

## Abbreviations used in this paper:

CEL: carboxyl ester lipase
ER: endoplasmic reticulum
MODY8: maturity onset diabetes of the young type 8
VNTR: variable number tandem repeats
16R: 16-repeat variable number tandem repeats
