# Supplementary material for: The MODY-Causing Mutation of the Human Carboxyl Ester Lipase Gene (CEL) Triggers Chronic Pancreatitis but not Diabetes in Mice
Source: Gastroenterology. Author manuscript; Available in PMC 2025 Jul 1. (PMC12185230; doi:10.1053/j.gastro.2025.01.243)
Supplement: 1 [file NIHMS2074059-supplement-1.pdf]

Supplementary Methods

Accession Numbers and Nomenclature

The accession number for the genomic sequence of the *Mus musculus* strain C57BL/6N carboxyl ester lipase (*Cel*) gene is MGP_C57BL6NJ_G0025943 (www.informatics.jax.org) and for the *Cel* cDNA sequence, NM_009885.2 (www.ncbi.nlm.nih.gov/nuccore). The accession number for the human *CEL* gene is ENSG00000170835 (www.ensembl.org). Regarding nomenclature, mouse carboxyl ester lipase (mCEL) refers to the mouse protein, CEL to the human protein, carboxyl ester lipase–maturity-onset diabetes of the young (CEL-MODY) to the human maturity-onset diabetes of the young, type 8 (MODY8) CEL protein, mCEL-MODY to mCEL containing the aberrant variable number tandem repeat (VNTR) repeats from CEL-MODY, and mCEL-16R to mCEL containing the 16 normal VNTR repeats from CEL.

Study Approvals

Mouse breeding, housing, and experiments were performed in parallel at the Laboratory Animal Facility, Faculty of Medicine, University of Bergen, Norway, and at Washington University, St Louis, Missouri. The study was approved by the Norwegian Animal Welfare Agency (FOTS IDs 13902 and 13510) and Institutional Animal Care and Use Committee at Washington University (IDs 19-1109 and 22-0356). Human pancreatic tissue samples were obtained from a biobank of pancreatic neoplastic lesions (ethical approval: REK Vest 2013/1772).[e1]

Expression of mCEL Variant Proteins in Cell Culture

The expression vector pcDNA3/mCEL–wild-type (WT) was engineered by subcloning the full-length mCEL coding sequence into the pcDNA3 vector using the HindIII/XhoI restriction sites. Partial mCEL coding sequences fused with either the human 16R VNTR sequence or the CEL-MODY VNTR sequence were synthesized by Genscript and used to replace the KpnI/XhoI fragment of pcDNA3/mCEL-WT to generate the constructs pcDNA3/mCEL-16R or pcDNA3/mCEL-MODY. Mutant constructs pcDNA3/mCEL-S194A and pcDNA3/mCEL-MODY-S194A were created through site-directed mutagenesis. All plasmids were confirmed by Sanger sequencing. Transfection of HEK293T cells, harvesting of media and cells, immunoblotting of CEL variant proteins, and lipase activity assays were performed according to established protocols.[e2]

Generation of Mouse Strains

Construction of the targeting vector and generation of the *Cel-MODY* and *Cel-16R* mice were performed by genOway by the same strategy and on the same genetic background (C57BL/6N; Charles River Laboratories) as previously described for the *Cel-HYB1* model.[e3] Briefly, the targeting vector consisted of a short arm (*Cel* exon 8-10 region), a middle arm (*Cel* exon 11 with humanized VNTR sequence, stemming either from the *CEL-MODY* or the *CEL-16R*^{allele})[e4] and a long arm (*Cel* exon 11 downstream sequence). C57BL/6N embryonic stem cells were electroporated with the vector and selected with G418. After molecular genetic screening (polymerase chain reaction, Southern blot), correctly recombined cell clones were injected into blastocysts and implanted into pseudopregnant females.

The *Cel-MODY* S194A and *Cel* S194A mice were generated on the genetic background of *Cel-MODY* and WT C57BL/6N (Charles River Laboratories) mice, respectively, disrupting the active serine residue (Ser194) in the catalytic site of mCEL.[e5,e6] Clustered regularly interspaced short palindromic repeats (CRISPR)/CRISPR-associated protein 9 genome editing technology was used to change the TCT codon for Ser194 to GCC encoding Ala by the Genome Engineering and iPSC Center (GEiC) at Washington University following standardized procedure as previously described.[e8]

Mouse Breeding and Maintenance

For breeding of heterozygous *Cel-MODY*, *Cel-16R,* and *Cel-MODY* S194A mice, we used heterozygous males and WT females. For generation of homozygous mice, breeding was performed with heterozygous males and females. The animals followed a normal 12-hour day/night cycle and were fed regular chow with ad libitum access to water and food. Both male and female mice were included in this study, except for homozygous *Cel-MODY* mice and 12-month-old mice (males only).

Initially, both WT littermates (*Cel*$^{+/+}$) and *Cel-16R* animals were used as controls. However, because growth and development were identical and histologic analyses demonstrated similar normal morphology for *Cel*$^{+/+}$ and *Cel-16R*, only *Cel*$^{+/+}$ animals were used as controls in some experiments. Each cohort had a minimum of six mice. Mouse genotyping was performed as previously described.[e3] Unique primer sequences were 5’- GCC AAA GAG ACA TGC AGT GAG AAG AGT ACC -3’ (forward) and 5’- CGA ATG TCA CAG CCC AGA ACT TCA GG -3’ (reverse) for *Cel-MODY* mice, and 5’- CCA CCA TGA GTC CAA TGA TTG CAC C -3’ (forward) and 5’- GGT GGC CTC CTG GTC GGT CAC T -3’ (reverse) for *Cel-16R* mice.

Organ Collection and Preparation of Tissues

At experimental endpoints, blood samples and tissues were harvested. All mice were sacrificed by carbon dioxide euthanasia, and blood was collected immediately by cardiac puncture. The tissues harvested were pancreas, liver, and epididymal adipose tissue. All tissue samples were either weighed and fixed in formaldehyde for histology/immunostaining analysis or snap-frozen in liquid nitrogen and stored at –80 °C for RNA, protein, and biochemical analysis.

UNCORRECTED PROOF

ARTICLE IN PRESS

Sodium Dodecyl Sulfate/ Polyacrylamide Gel Electrophoresis and Immunoblotting

Preparation of tissue samples, sodium dodecyl sulfate/ polyacrylamide gel electrophoresis and immunoblotting were performed as previously described.[e3] Primary antibodies were rabbit anti-CEL (1:10,000)[e7] and mouse anti–β-actin (Sigma, A5441; 1:2,000).

Histology and Evaluation of Pathology and Enzyme Activity Assays

Histology, trichrome staining, evaluation of pathology, transmission electron microscopy, and measurements of hydroxyproline level, amylase, lipase, and trypsin activities were conducted as previously described.[e3,e8]

Immunostaining

Chromogenic (CEL, F4/80) and immunofluorescence (binding immunoglobulin protein, insulin, glucagon, Ki67) staining were performed as previously described.[e3,e9] Primary antibodies were anti-CEL (Sigma-Aldrich, HPA052701; 1:200), anti-F4/80 (Thermo Fisher Scientific, #14480182; 1:200), anti–binding immunoglobulin protein (Abcam, ab21685; 1:400), anti-insulin (Abcam, #ab195956; 1:400), anti-glucagon (Sigma-Aldrich, # G2654, 1:800), and anti-Ki67 (Abcam, ab15580, 1:400). Stained tissue sections were scanned and virtual slide image (vsi) files from Olympus VS120 S6 slide scanner (EVIDENT) were acquired with 20 × UPlanSApo NA 0.75 and analyzed by QuPath (version 0.5.1).[e10]

Assessment of Endoplasmic Reticulum Stress Markers

RNA extraction, reverse transcription, and quantitative real-time polymerase chain reaction were performed as previously described.[e8] Expression of mRNA was calculated using the ΔΔCT method, and the results were presented as fold change calculated with the formula $2^{-\Delta\Delta CT}$.

Measurements of Pancreatic Endocrine Function

Mice were fasted for 4 hours for insulin tolerance testing (ITT) and overnight (14-16 hours) for glucose tolerance testing (GTT) and insulin measurements. GTT, ITT, and insulin measurements were performed as described in St Louis et al.[e11] Heterozygous male mice at 6 and 12 months received 1 unit of insulin/kg body weight. Heterozygous females and all homozygous mice were given 0.75 units of insulin/kg body weight.

Magnetic Resonance Imaging

Magnetic resonance imaging was performed using a small-animal 7 Tesla magnetic resonance imaging–positron emission tomography scanner (MR Solutions) as described previously.[e12] T1- and T2-weighted images were acquired coronally with echo time/repetition time 11/1177 ms and 45/3500 ms, respectively, and with three averages, 192 × 256 matrix size, 0.5 mm slice thickness, 0.6 mm slice gap, and 0.156 × 0.156 mm resolution.

Statistics

Results were plotted either as individual data points or as mean value. Either standard deviation or standard error of the mean was presented. Differences of means were analyzed by a two-tailed unpaired Student t test using Microsoft Excel (between two groups) and by one-way analysis of variance mixed-effects model with Dunnett's multiple comparisons test (more than two groups). $P < .05$ was considered statistically significant.

References

e1. El Jellas K, et al. Cancer Med 2017;6:1531–1540.
e2. Cassidy BM, et al. Hum Mutat 2020;41:1967–1978.
e3. Fjeld K, et al. Pancreatology 2022;22:1099–1111.
e4. Ræder H, et al. Nat Genet 2006;38:54–62.
e5. DiPersio LP, et al. J Biol Chem 1990;265:16801–16806.
e6. **Lidmer AS, Kannius M**, et al. Genomics 1995; 29:115–122.
e7. Xiao X, et al. J Biol Chem 2016;291:23224–23236.
e8. Zhu G, et al. Gut 2023;72:1340–1354.
e9. Kahraman S, et al. Nat Metab 2022;4:76–89.
e10. Bankhead P, et al. Sci Rep 2017;7:16878.
e11. St Louis JL, et al. J Biol Chem 2023;299:104986.
e12. Espedal H, et al. Front Oncol 2024;14:1334541.

Author names in bold designate shared co-first authorship.

UNCORRECTED PROOF

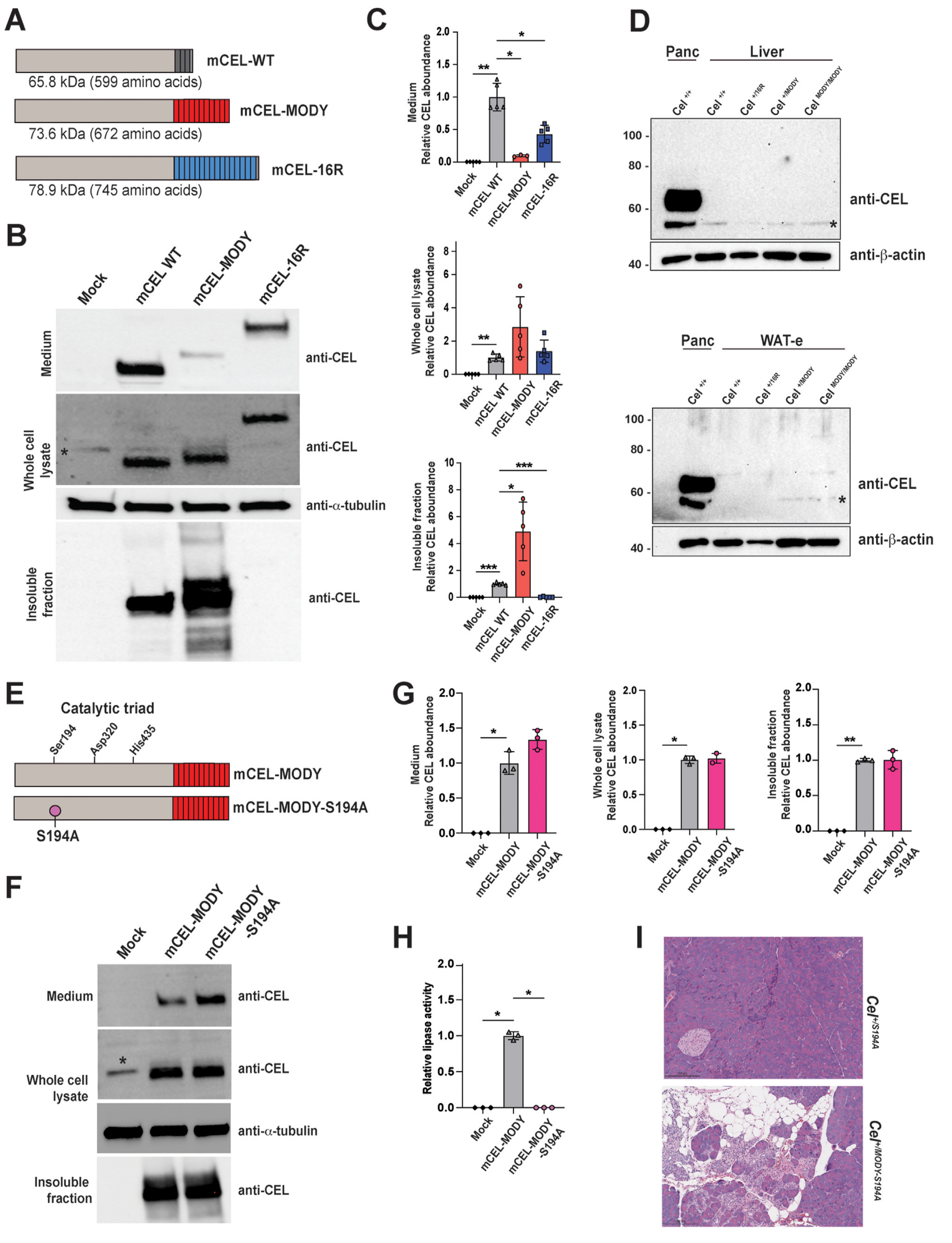
A
mCEL-WT
65.8 kDa (599 amino acids)
mCEL-MODY
73.6 kDa (672 amino acids)
mCEL-16R
78.9 kDa (745 amino acids)
B
Mock
mCEL WT
mCEL-MODY
mCEL-16R
Medium
Whole cell lysate
Insoluble fraction
anti-CEL
anti-α-tubulin
C
Medium Relative CEL abundance
Whole cell lysate Relative CEL abundance
Insoluble fraction Relative CEL abundance
D
Panc
Liver
WAT-e
anti-β-actin
E
Catalytic triad
Ser194
Asp320
His435
mCEL-MODY
mCEL-MODY-S194A
S194A
F
G
H
Relative lipase activity
I
Cel+/S194A
Cel+/MODY-S194A

web 4C/FPO

UNCORRECTED PROOF

Supplementary Figure 1. Experimental evaluation of humanized carboxyl ester lipase (Cel) constructs. *(A)* Schematic presentation of tested mouse carboxyl ester lipase (mCEL) constructs: (i) mCEL–wild-type (WT) corresponds to endogenous mouse Cel (three variable number tandem repeats expressed by $Cel^{+/+}$ mice. (ii) mCEL–maturity-onset diabetes of the young (MODY) contains the aberrant repeats of the original MODY family[2] and is expressed by $Cel^{+/MODY}$ mice. (iii) mCEL–16-repeat (16R) contains the normal 16 repeats of human CEL[2] and is expressed by $Cel^{+/16R}$ mice. *(B,C)* Effect of the two human CEL VNTR regions on the properties of mCEL. HEK 293T cells were transfected to express mCEL-WT, mCEL-MODY, or mCEL-16R. Sixty-eight hours post-transfection, conditioned media and transfected cells were harvested. *(B)* Representative immunoblots of CEL protein in the medium, cell lysates, and the insoluble pellet fraction of transfected cells. α-tubulin expression serves as loading control. *(C)* Quantification of CEL protein amount relative to that in mCEL-WT for the medium, lysate, and insoluble fraction in *B* (n = 3 to 5). *(D)* Western blot of lysates from liver (upper panel) and epididymal white adipose tissue (WAT-e; lower panel) of mice (age 3 months) with different genotypes: $Cel^{+/+}$, $Cel^{+/16R}$, $Cel^{+/MODY}$ and $Cel^{MODY/MODY}$. One representative lysate for each genotype is shown. For each blot, a positive control lysate from $Cel^{+/+}$ pancreas was run in the left lane. Beta-actin expression serves as loading control. *(E-I)* Effect of the S194A-mutation on mCEL-MODY. *(E)* Schematic presentation of the mCEL-MODY protein without and with the inactivating mutation S194A within the catalytic triad. *(F)* Expression of mCEL-MODY proteins in transfected HEK293T cells. Sixty-eight hours post-transfection, conditioned media and transfected cells were harvested and processed for analysis. A representative immunoblot of mCEL-MODY and mCEL-MODY-S194A proteins in the medium, whole cell lysate, and insoluble fraction is shown. α-tubulin expression serves as loading control. *(G)* Quantification of mCEL-MODY-S194A protein amount relative to that in mCEL-MODY for medium, lysate, and insoluble fraction in *F* (n = 3). *(H)* Relative lipase activity in extracts from transfected HEK 293T cells expressing the mCEL protein variants of panel *E*. *(I)* Representative hematoxylin and eosin–stained pancreas sections of $Cel^{+/S214A}$ and $Cel^{+/MODY\text{-}S194A}$ mice at age 6 months. Scale bars are 200 μm. In *C, G* and *H*, individual values with mean (horizontal bar) $\pm$ SD are shown. The asterisks in *B, D,* and *F* indicate unspecific signals previously observed for the anti-CEL antibody in cellular lysates.[e4] *$P \le .05$. **$P \le .001$. ***$P \le .0001$. Panc, ; .

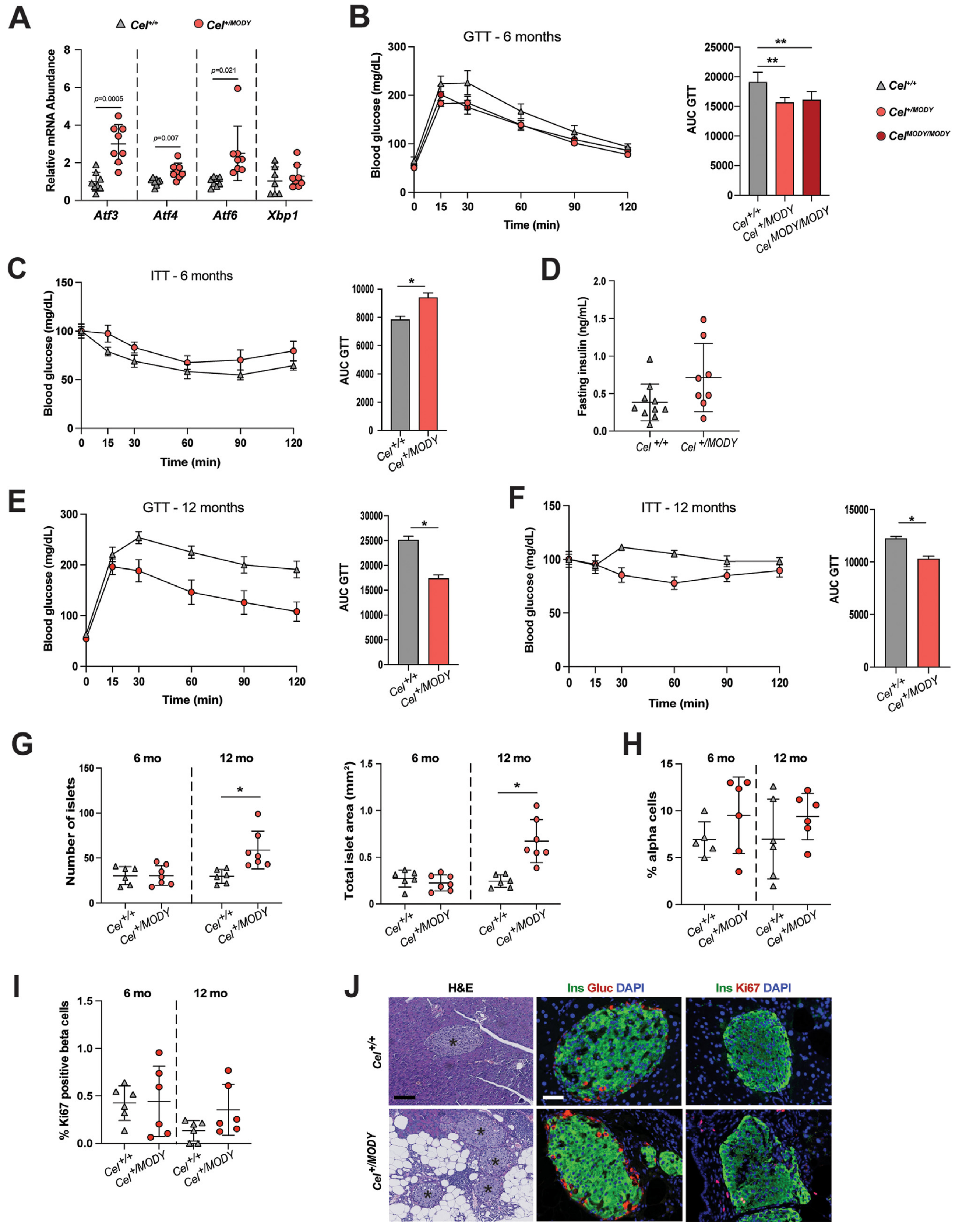
A
Cel+/+
Cel+/MODY
Relative mRNA Abundance
p=0.0005
p=0.007
p=0.021
Atf3
Atf4
Atf6
Xbp1
B
GTT - 6 months
Blood glucose (mg/dL)
Time (min)
AUC GTT
**
**
Cel+/+
Cel +/MODY
Cel MODY/MODY
CelMODY/MODY
C
ITT - 6 months
Blood glucose (mg/dL)
Time (min)
AUC GTT
*
D
Fasting insulin (ng/mL)
Cel +/+
Cel +/MODY
E
GTT - 12 months
Blood glucose (mg/dL)
Time (min)
AUC GTT
*
F
ITT - 12 months
Blood glucose (mg/dL)
Time (min)
AUC GTT
*
G
6 mo
12 mo
Number of islets
Total islet area (mm2)
*
H
% alpha cells
I
% Ki67 positive beta cells
J
H&E
Ins Gluc DAPI
Ins Ki67 DAPI
Cel+/+
Cel+/MODY

web 4C/FPO

UNCORRECTED PROOF

Supplementary Figure 2. Endoplasmic reticulum **(**ER) stress, endocrine function and endocrine cell content in the carboxyl ester lipase (*Cel*)–maturity-onset diabetes of the young (*MODY*) mouse. *(A)* mRNA levels of ER stress markers *Atf3, Atf4,* and *Atf6 og Xbp1* in $Cel^{+/+}$ and $Cel^{+/MODY}$ mice at age 6 months. Individual values with mean (horizontal bar) ± SD. (*B-F*) Assessment of endocrine function. Intraperitoneal glucose and insulin tolerance tests (GTT/ITTs) were performed in mice at age 6 *(B, C)* and 12 *(E, F)* months. Data are shown as mean ± SEM. Corresponding calculated areas under the curve (AUC) are presented to the right of each panel with data shown as mean ± SD. *(D)* Fasting insulin levels in serum from mice at age 6 months. Individual values are shown with mean ± SD. *(G)* Quantification of islets in pancreas sections stained (chromogenic) for insulin. The left panel shows the number of islets per pancreatic section. Right panel presents the total islet area per pancreatic section. *(H)* Quantification of glucagon-positive cells in islets based on immunofluorescent co-staining for insulin and glucagon. Data are shown as % alpha cells of total number of islet cells. *(I)* Quantification of beta-cell proliferation in islets based on immunofluorescence co-staining for proliferation marker Ki67 and insulin. Data are shown as % Ki67 positive beta-cells. For *G-I*, n = 6 to 7 mice, at age 6 and 12 months (mo), were assessed. Individual values are shown with mean ± SD. *(J)* Representative images of pancreatic hematoxylin and eosin (H&E)–stained sections and immunofluorescent co-staining for insulin/glucagon or insulin/Ki67 in $Cel^{+/+}$ and $Cel^{+/MODY}$ mice at age 12 months. Asterisks indicate islets in the left panel. Scale bars are 100 μm (H&E) and 40 μm (immunostainings). Ins, insulin; Gluc, glucagon; DAPI, 4,6-diamidino-2 phenylindole nuclear staining. *$P \leq .05$. **$P \leq .001$. ***$P \leq .0001$.
